# Co-transfer of tumor-specific effector and memory CD8+ T cells enhances the efficacy of adoptive melanoma immunotherapy in a mouse model

**DOI:** 10.1186/s40425-018-0358-2

**Published:** 2018-05-29

**Authors:** Amanda Contreras, Megan V. Beems, Andrew J. Tatar, Siddhartha Sen, Prakrithi Srinand, M. Suresh, Tahra K. Luther, Clifford S. Cho

**Affiliations:** 1 Department of Pathobiological Sciences, University of Wisconsin School of Veterinary Medicine, 2015 Linden Drive, Madison, WI 53706 USA; 2 Department of Surgery, University of Wisconsin School of Medicine and Public Health, 600 Highland Avenue, Madison, WI 53792 USA; 3 Department of Pathobiological Sciences, University of Wisconsin School of Veterinary Medicine, 2015 Linden Drive, Madison, WI 53706 USA; 4 VA Ann Arbor Healthcare System, 2215 Fuller Road, Ann Arbor, MI 48105 USA; 5 Department of Surgery, University of Michigan Medical School, 1500 East Medical Center Drive, Ann Arbor, MI 48109 USA

**Keywords:** Adoptive transfer, Immunotherapy, T cell, Memory, Effector, Melanoma, Cancer

## Abstract

**Background:**

Adoptive cell transfer (ACT) is a promising cancer immunotherapeutic strategy that remains ineffective for a large subset of patients. ACT with memory CD8+ T cells (T_mem_) has been shown to have superior efficacy compared to traditional ACT with effector CD8+ T cells (T_eff_). T_eff_ and T_mem_ have complementary physiological advantages for immunotherapy, but previous publications have not examined ACT using a combination of T_eff_ and T_mem_.

**Methods:**

Splenocytes harvested from Ly5.1+/C57BL/6 mice during and after infection with lymphocytic choriomeningitis virus (LCMV) were used to generate bona fide effector and memory CD8+ T cells specific for the LCMV epitope peptide GP33. Congenic Ly5.2+/C57BL/6 mice were inoculated with B16F10 melanoma cells transfected to express very low levels of GP33, then treated with ACT 7 days later with GP33-specific T_eff_, T_mem_, or a combination of T_eff_ + T_mem_.

**Results:**

Inhibition of melanoma growth was strongest in mice receiving combinatorial ACT. Although combinatorial ACT and memory ACT resulted in maximal intratumoral infiltration of CD8+ T cells, combinatorial ACT induced stronger infiltration of endogenous CD8+ T cells than T_mem_ ACT and a stronger systemic T cell responsiveness to tumor antigen. In vitro assays revealed rapid but transient melanoma inhibition with T_eff_ and gradual but prolonged melanoma inhibition with T_mem_; the addition of T_mem_ enhanced the ability of T_eff_ to inhibit melanoma in a manner that could be reproduced using conditioned media from activated T_mem_ and blocked by the addition of anti-IL-2 blocking antibody.

**Conclusions:**

These findings suggest that a novel combinatorial approach that takes advantage of the unique and complementary strengths of tumor-specific T_eff_ and T_mem_ may be a way to optimize the efficacy of adoptive immunotherapy.

## Background

Adoptive cell transfer (ACT) is a promising strategy for cancer immunotherapy that involves the isolation, expansion, and infusion of tumor-specific CD8+ T cells. When combined with interleukin-2 (IL-2) and lymphodepletion, ACT has led to complete and durable tumor regression in up to 20% of patients with metastatic melanoma [1]. Despite this, ACT remains ineffective for a large subset of patients.

Traditionally, ACT involves the use of terminally differentiated CD8+ effector T cells (T_eff_) collected from tumor-infiltrating lymphocytes (TILs). When naïve T cells encounter antigen, they clonally expand and differentiate into T_eff_ that have the ability to rapidly clear cells expressing their cognate antigen [2, 3]. Following antigen clearance, the majority of T_eff_ then undergo apoptosis, while a small population of antigen-specific CD8+ memory T cells (T_mem_) persists long-term and can rapidly proliferate upon antigen re-exposure [2, 3].

The use of different T cell subsets is one potential way to improve the efficacy of ACT. Less-differentiated effector cells have been shown to have superior antitumor immunity compared to terminally-differentiated cells, potentially due to a number of mechanisms such as decreased IL-2 production and increased apoptosis with further differentiation [4, 5]. Our laboratory and others have also demonstrated that ACT with T_mem_ is more effective than ACT with T_eff_ or naïve T cells [6, 7]. T_mem_ appear to be uniquely resistant to melanoma-induced suppression and generate a stronger intratumoral immune response [7, 8].

No previous publications have explored performing ACT with a combination of T_mem_ and T_eff_. Although T_eff_ are prone to apoptosis, we hypothesized that this limitation could be offset by the innate proliferative ability of T_mem_, with T_eff_ contributing to initial tumor control and T_mem_ to later tumor control. In a murine model, we compared the efficacy of ACT using bona fide T_eff_, T_mem_, and a combination of both. We utilized a novel melanoma tumor expressing very low levels of a viral peptide in order to dissect the biological effects of peptide-specific CD8+ T cells of true effector and memory differentiation. This methodology enables us to examine the therapeutic implications of bona fide effector and memory tumor-specific CD8+ T cells in a manner not possible with standard murine melanoma targets like GP100. Using in vitro experiments, we explored the temporal cell killing patterns and paracrine effects of these T cell subsets. We discovered that the combination of T_eff_ + T_mem_ led to the strongest control of melanoma tumor growth, potentially due to complementary cell killing patterns and local production of IL-2 by T_mem_.

## Methods

### Mice

Seven- to eight-week old female congenic C57BL/6 mice with allele Ly5.1+ (T cell donor mice) or allele Ly5.2+ (T cell recipient mice) were purchased from Taconic (Hudson, NY) and maintained in sterile housing. All mouse work was reviewed and approved by the University of Wisconsin and William S. Middleton Memorial VA Hospital and VA Ann Arbor Healthcare Animal Care and Use Committees.

### Tumor cell lines and effector/memory cell generation

Viral infections and tumor inoculations were performed per our previously published protocols [7, 9, 10]. In brief, Ly5.1+/C57BL/6 mice were infected with 2 × 10^5^ PFU of Armstrong strain lymphocytic choriomeningitis virus (LCMV; gift from Marulasiddappa Suresh, University of Wisconsin) via intraperitoneal injection to generate CD8+ T cells specific for GP33, a class I MHC-restricted LCMV surface glycoprotein. Splenocytes were harvested 8 days after infection (at which time LCMV peptide-specific CD8+ T cells are of effector differentiation) to obtain bona fide T_eff_ (as evidenced by CD127^low^/KLRG1^high^ expression) and 50–80 days after infection (at which time LCMV peptide-specific CD8+ T cells are of memory differentiation) to obtain bona fide T_mem_ (as evidenced by CD127^high^/KLRG1^low^ expression) [7, 8]. The yield of GP33-specific memory CD8+ T cells was increased by adoptively transferring Ly5.1+/C57BL/6 mice with 10^3^ Ly5.1+/CD8+ T cells derived from splenocytes of Ly5.1+/P14 TCR transgenic mice (C57BL/6 background mice with CD8+ T cell specificity for GP33 [7]. The spontaneous mouse melanoma cell line B16F10 was transfected with a plasmid encoding GP33, and poorly immunogenic clones with low levels of GP33 expression were selected to create a new B16GP33 cell line, which we have previously demonstrated to be of comparably low immunogenicity as parental B16F10 [9, 10]. This line was cultured in RPMI-1640 medium (Mediatech, Herndon, VA) with 10% fetal bovine serum, 2 mM L-glutamine, 100 μg/mL streptomycin, and 100 U/mL penicillin (Life Technologies, Inc., Grand Island, NY). Ly5.2+/C57BL/6 mice received subcutaneous injections of 1 × 10^6^ B16GP33 cells suspended in serum-free RPMI-1640 to generate flank melanoma tumors.

### Adoptive cell transfer

Adoptive cell transfer was performed per our previously published protocol [7, 8, 10]. As previously observed, GP33-specific CD8+ T cells typically comprised approximately 8–10% of splenocytes harvested from effector and memory T cell donor mice [7, 8]. After obtaining splenocytes from mice infected with LCMV, magnetic bead separation columns (Miltenyi, Auburn, CA) were used to isolate CD8+ T cells. Flow cytometry was performed to quantify the percentage of tumor-specific GP33-specific CD8+ T cells. 10^5^ GP33-specific CD8+ T_eff_, T_mem_, or 5 × 10^4^ T_eff_ + 5 × 10^4^ T_mem_ were adoptively transferred via retro-orbital intravenous injections into B16F10- or B16GP33 melanoma-bearing mice at specific time points after tumor inoculation.

### MTT assay

For multiple timepoints, a total number of 2 × 10^4^ GP33-specific CD8+ T_eff_ and T_mem_ or both were separately co-cultured with 2 × 10^4^ B16GP33 melanoma cells at an overall effector:target ratio of 1:1 in 48-well plates in the presence of 10 ng/mL recombinant human IL-2 (BD Biosciences, San Jose, CA) for MTT assays as previously described [7]. T cells were characterized and isolated using the methodology described for adoptive cell transfer. Viable B16GP33 melanoma cells were quantified using the Celltiter 96 non-radioactive MTT assay kit (Promega, Madison, WI) following the manufacturer’s instructions.

### Conditioned media assay

For 5 h, GP33-specific CD8+ T_eff_ and T_mem_ were cultured separately or together in RPMI-1640 with 10 ng/mL IL-2 and 0.1 μg/mL GP33 peptide (gift from Marulasiddappa Suresh, University of Wisconsin). Conditioned media was collected by centrifuging wells and collecting the supernatant. For 12 h, GP33-specific CD8+ T_eff_ and T_mem_ were then co-cultured with B16GP33 melanoma cells with or without conditioned media and anti-IL-2 (BD Biosciences, San Jose, CA) for MTT assays as described above.

### Flow cytometry

Flow cytometric analysis was performed per our previously published protocol [7–10]. Once harvested, tumor samples were processed into single-cell suspensions with mechanical fractionation on a fine wire mesh followed by lymphocyte isolation using Histopaque (Sigma Aldrich, St. Louis, MO). Splenocytes before ACT and tumor-infiltrating lymphocytes (TIL) 4 to 14 days after ACT were stained with APC-labeled MHC class I tetramers loaded with GP33 peptide (NIH Tetramer Facility Core, Atlanta, GA), PerCP-labeled anti-CD8 mAb, PE-labeled anti-Ly5.1 mAb, and FITC-labeled anti-CD44 mAb (BD Biosciences, San Jose, CA). Intracellular staining required stimulation of lymphocytes with media alone or with GP33 peptide (1 μg/mL), brefeldin A, and recombinant human IL-2 (10 ng/mL) at 37°C for 5 h. A BD LSRFortessa flow cytometer (BD Biosciences, San Jose, CA) was used and data were analyzed using FlowJo software (Tree Star Inc., Ashland, OR).

### Statistical analysis

Experimental data was analyzed using IBM SPSS statistical software version 23 (Armonk, NY). T-test was used to compare groups and a repeated-measures analysis of variance (ANOVA) with pair-wise comparisons performed using Fisher’s protected least significant difference tests were used to compare multiple groups. Significance was defined as *p* < 0.05, and error bars represent standard errors of the mean.

## Results

### Co-ACT of T_eff_ + T_mem_ is more effective at inhibiting in vivo melanoma growth than ACT of either T cell subset alone

**Fig. 1:**
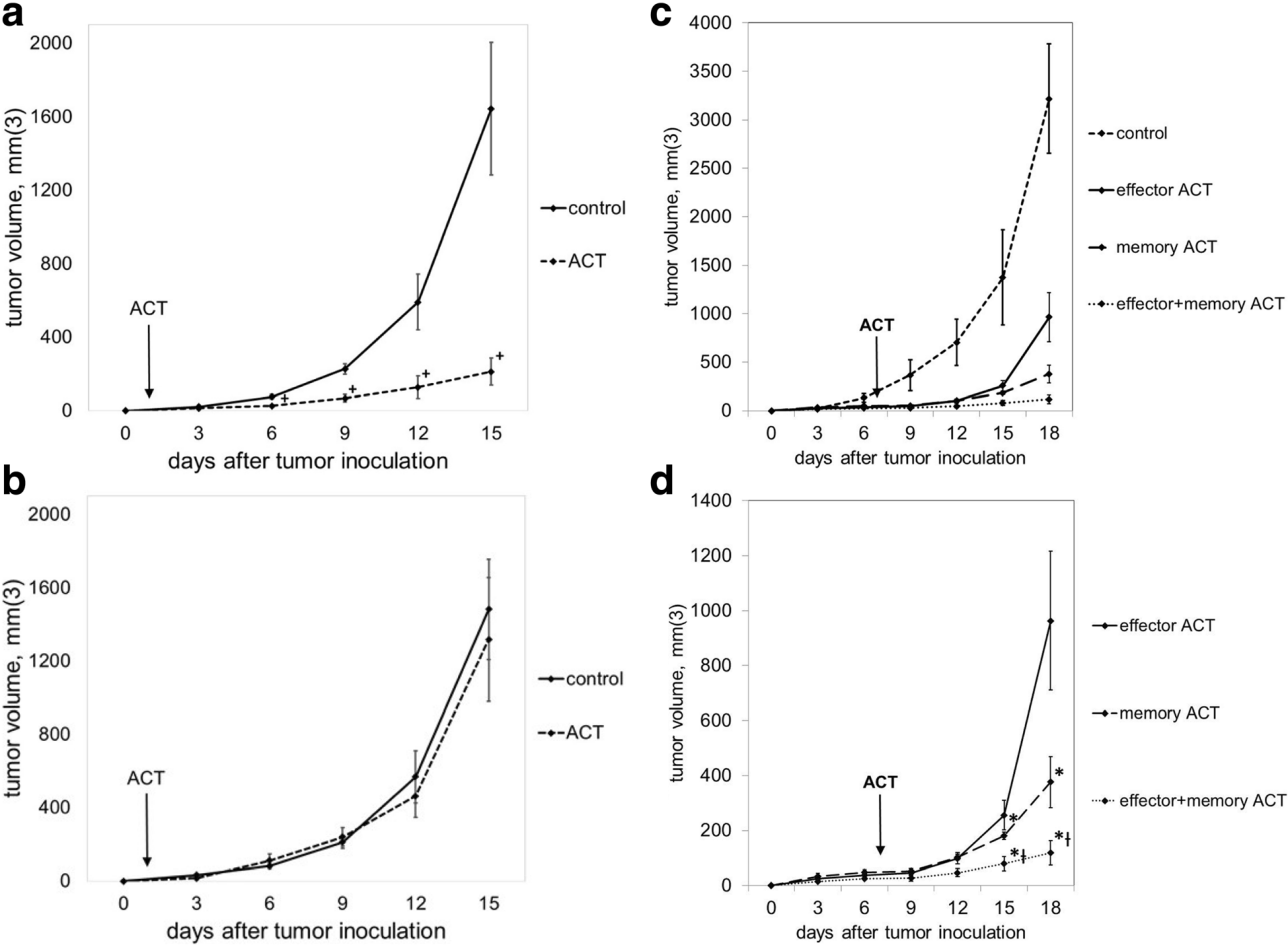
ACT with a combination of T_eff_ + T_mem_ resulted in optimal control of melanoma growth. Mice bearing B16GP33 (**a**) or B16F10 (**b**) melanoma were untreated (control) or treated with ACT of 10^5^ GP33-specific CD8+ T cells one day after tumor inoculation. Serial tumor measurements confirmed that ACT resulted in a GP33-specific inhibition of melanoma tumor growth. (**c**) Mice bearing B16GP33 melanoma were treated 7 days after tumor inoculation with no treatment (control) or 10^5^ T_eff_, 10^5^ T_mem_, or 5 × 10^4^ T_eff_ + 5 × 10^4^ T_mem_. Comparison of treated groups (**d**) demonstrates optimal control of melanoma growth with combinatorial ACT. This experiment was repeated three times with similar results, 4–5 mice per group. (**+** *p* < 0.05 compared with control; * *p* < 0.05 compared with effector ACT; **Ɨ** *p* < 0.05 compared with memory ACT)

To verify the peptide specificity of our ACT model, C57BL/6 mice were treated with 10^5^ GP33-specific T_eff_ one day after subcutaneous inoculation with B16GP33 melanoma or parental B16F10 melanoma (which does not express GP33). As shown in Fig. 1a-b, early ACT strongly suppressed the growth of B16GP33 melanoma but not B16F10 melanoma, suggesting that the effect of ACT was peptide-specific. Moreover, the kinetics of B16GP33 tumor growth in immunocompetent mice was comparable to those of parental B16F10 tumor. To compare the efficacy of ACT with different T cell subsets, Ly5.2+/C57BL/6 mice received ACT of 10^5^ GP33-specific CD8+ T_eff_, T_mem_, or T_eff_ + T_mem_ on day 7 after subcutaneous inoculation with 10^6^ B16GP33 cells. As shown in Fig. 1c-d, memory ACT resulted in significantly greater inhibition of tumor growth compared to effector ACT as previously described.^7^ Combinatorial ACT with T_eff_ + T_mem_ had significantly greater inhibition of tumor growth compared to ACT with T_eff_ or T_mem_ separately, making this combination the most effective strategy for ACT.

### Co-ACT of T_eff_ + T_mem_ promotes more potent intratumoral CD8 + T cell infiltration than ACT of either T cell subset alone

**Fig. 2:**
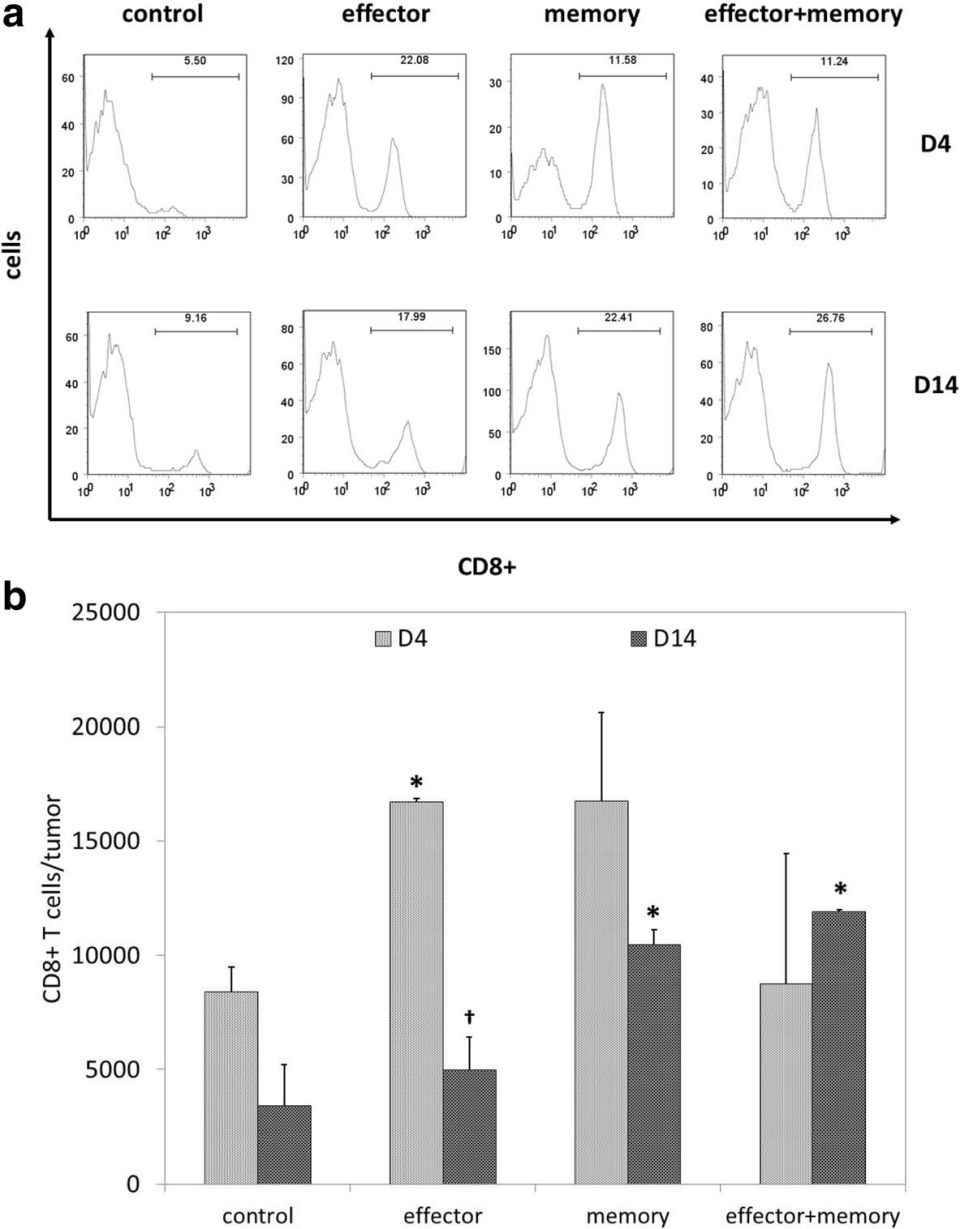
Combinatorial ACT results in a stronger intratumoral T cell response. Seven days after tumor inoculation, Ly5.2+ mice were treated with 10^5^ T_eff_, 10^5^ T_mem_, or 5 × 10^4^ T_eff_ + 5 × 10^4^ T_mem_ derived from Ly5.1+ mice. TIL were harvested from melanoma tumors resected 4 or 14 days after ACT and stained for flow cytometric analysis. (**a**) Representative TIL data (gated on lymphocyte populations) demonstrate that CD8+ T cells comprised the highest percentage of TIL after effector ACT on day 4 and after combinatorial ACT on day 14. (**b**) Overall comparison of total numbers of intratumoral CD8+ T cells on days 4 and 14 demonstrated a transient increase after effector ACT and a sustained increase after memory ACT and combinatorial ACT. This experiment was repeated twice with similar results, 4–5 mice per group. (* *p* < 0.05 compared with control condition; **†** *p* < 0.05 compared with day 4)

To determine if the efficacy of combinatorial ACT was associated with stronger intratumoral T cell infiltration, mice were euthanized 4 and 14 days after ACT to allow for tumor harvesting and flow cytometric analysis of TIL populations. Representative data shown in Fig. 2a demonstrate that CD8+ T cells comprised a higher percentage of TIL in mice treated with ACT in comparison with untreated controls. As shown in Fig. 2b, total numbers of intratumoral CD8+ were transiently elevated on day 4 in mice treated with effector ACT, but this effect was short-lived; in contrast, intratumoral CD8+ T cells remain elevated on day 14 in mice treated with memory and combinatorial ACT.

### The more potent intratumoral CD8+ T cell infiltration seen with combinatorial ACT is the result of a stronger endogenous immune response

**Fig. 3:**
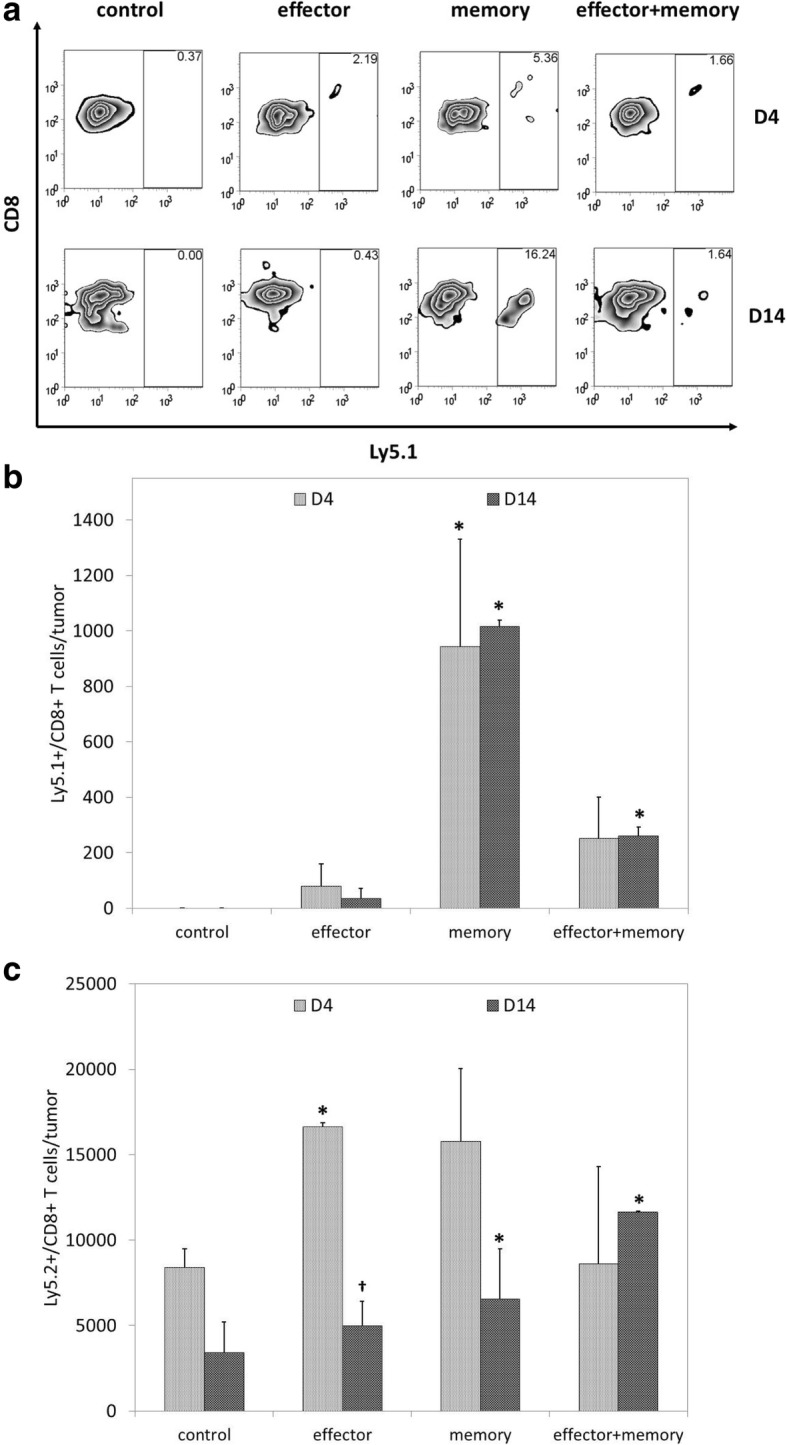
The stronger local CD8+ T cell response seen with combinatorial ACT is largely the result of endogenous T cell recruitment. Seven days after tumor inoculation, Ly5.2+ mice were treated with 10^5^ T_eff_, 10^5^ T_mem_, or 5 × 10^4^ T_eff_ + 5 × 10^4^ T_mem_ derived from Ly5.1+ mice. Melanoma tumors were resected 4 or 14 days after ACT and TIL were harvested for flow cytometric analysis. (**a**) Representative data demonstrate higher percentages of Ly5.1+/CD8+ T cells in tumors after memory and combinatorial ACT. (**b**) Analysis of total numbers of Ly5.1+/CD8+ T cell populations showed that significant and durable infiltration of adoptively transferred cells was observed after memory ACT; combinatorial ACT, which used half the number of adoptively transferred T_mem_ as memory ACT, resulted in smaller numbers of intratumoral Ly5.1+/CD8+ T cells. (**c**) Analysis of total numbers of Ly5.2+/CD8+ T cell populations showed that memory ACT resulted in a significant recruitment of endogenously-derived CD8+ T cells that persisted on day 14; despite using half the number of T_mem_, combinatorial ACT also resulted in durable infiltration of endogenously-derived CD8+ T cells. This experiment was repeated twice with similar results, 4–5 mice per group. (* *p* < 0.05 compared with control condition; **†** *p* < 0.05 compared with day 4)

To characterize the increase in intratumoral CD8+ T cell infiltration seen following ACT, flow cytometric analysis was used to quantify the presence of Ly5.1+ adoptively transferred CD8+ T cells and Ly5.2+ endogenous CD8+ T cells. Representative flow cytometric data are shown as percentages in Fig. 3a; overall data are shown as total cell numbers in Fig. 3b-c. As shown in Fig. 3b, Ly5.1+ adoptively transferred CD8+ T cells comprised a small and short-lived fraction of TIL after effector ACT but were present in substantial and stable numbers after memory ACT; combinatorial ACT, which used half the number of T_mem_ used in memory ACT, resulted in smaller numbers of intratumoral Ly5.1+/CD8+ TIL. In contrast, as shown in Fig. 3c, combinatorial ACT resulted in the strongest intratumoral infiltration of endogenous CD8+ T cells.

### Combinatorial ACT results in a stronger systemic CD8+ T cell response against tumor antigen

**Fig. 4:**
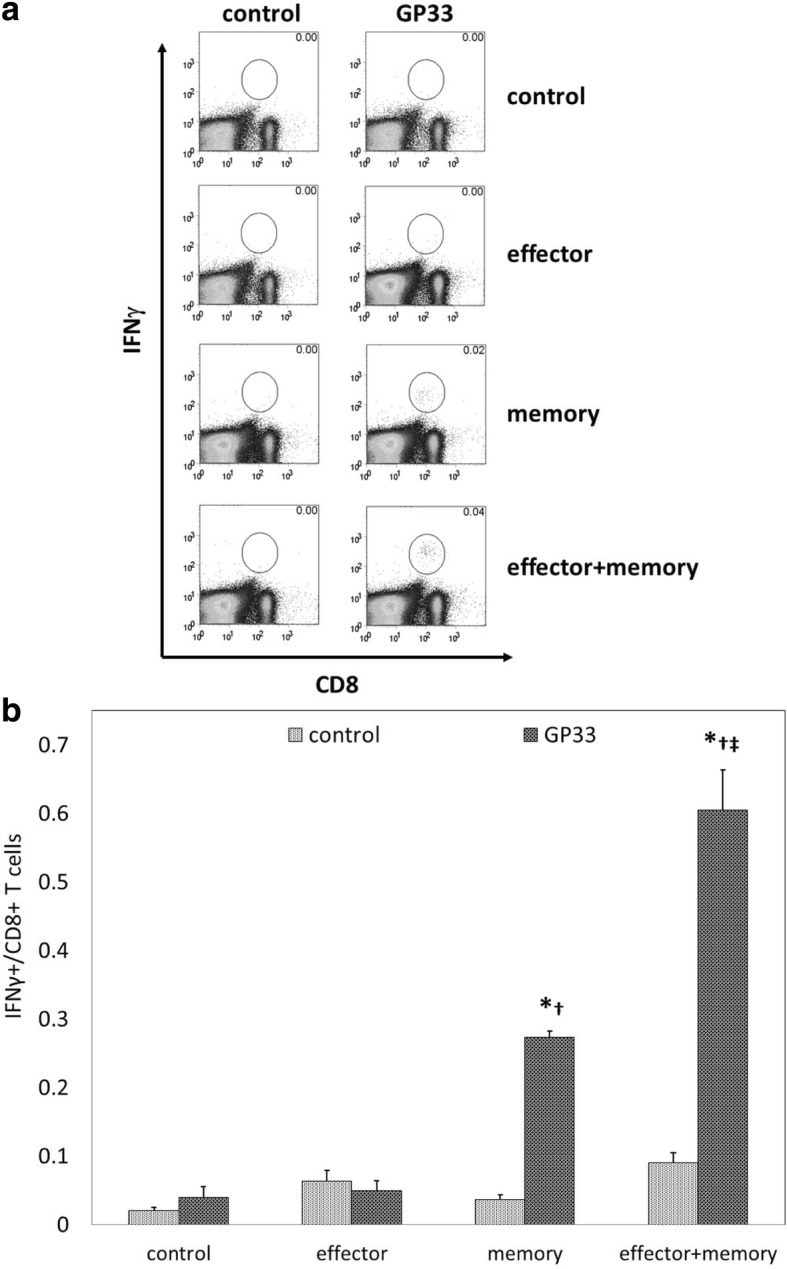
Combinatorial ACT results in the strongest level of systemic CD8+ T cell responsiveness to tumor antigen. Seven days after tumor inoculation, Ly5.2+ mice were treated with 10^5^ T_eff_, 10^5^ T_mem_, or 5 × 10^4^ T_eff_ + 5 × 10^4^ T_mem_ derived from Ly5.1+ mice. Spleens were resected 14 days after ACT and splenocytes were incubated with media alone (control) or with tumor antigen (GP33). (**a**) Representative data demonstrate negligible levels of IFNγ expression by splenocytes in response to GP33 stimulation after no ACT or effector ACT, with increasing levels seen after memory ACT and combinatorial ACT, respectively. (**b**) The highest levels of IFNγ expression by splenocytes in response to GP33 stimulation were seen after combinatorial ACT. This experiment was repeated with similar results, 4 mice per group. (* *p* < 0.05 compared with control condition; **†** *p* < 0.05 compared with effector ACT, **‡** *p* < 0.05 compared with memory ACT)

To determine if combinatorial ACT improved systemic antitumor responsiveness in addition to local antitumor activity, splenocytes harvested 14 days after ACT were stimulated with or without GP33 peptide in the presence of IL-2 and brefeldin A, and stained to assess intracellular levels of IFNγ expression. Representative flow cytometric data are shown as percentages in Fig. 4a; overall data are shown as total cell numbers in Fig. 4b. As shown in Fig. 4b, memory ACT resulted in stronger splenocyte reactivity to GP33 than effector ACT, as we have previously described.^7^ However, combinatorial ACT produced significantly greater IFNγ expression among splenocytes than memory ACT, suggesting that combinatorial ACT may induce a much stronger level of CD8+ T cell responsiveness to tumor antigen.

### Combinatorial ACT may benefit from temporal differences in melanoma cell killing between T_eff_ and T_mem_

**Fig. 5:**
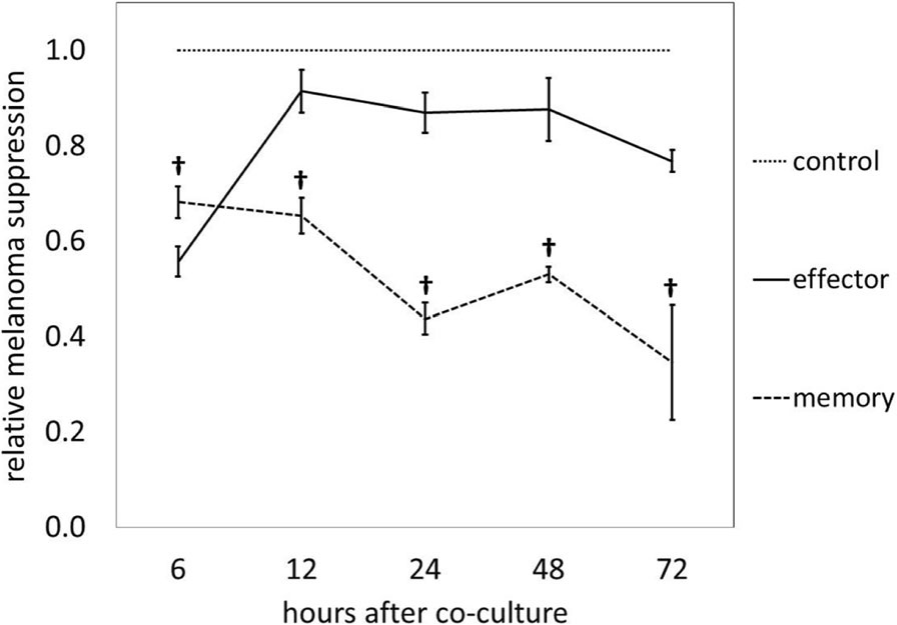
T_mem_ and T_eff_ have different temporal patterns of melanoma inhibition in vitro. T_eff_ and T_mem_ were co-cultured with B16GP33 melanoma cells in the presence of 10 ng/mL recombinant human IL-2 for 72 h. Control wells contained no T cells. Serial MTT assays were performed every 6 h. Over approximately the first 8 h, T_eff_ produced significantly greater inhibition of B16GP33. At all later time points, T_mem_ demonstrated significantly greater suppression of tumor growth. This experiment was repeated twice with similar results, 3 samples per group. (**†** *p* < 0.05 compared with T_eff_)

To further explore the improved efficacy of combinatorial ACT, T_eff_ and T_mem_ were co-cultured in vitro with B16GP33 melanoma cells in the presence of IL-2 for 72 h. As shown in Fig. 5, serial MTT assays demonstrated that T_eff_ inhibited melanoma more potently than T_mem_ at 6 h. However, whereas the ability of T_eff_ to inhibit melanoma was lost at later timepoints, T_mem_ continued to inhibit melanoma for the duration of time points tested (up to 72 h), suggesting that the enhanced therapeutic efficacy of combinatorial ACT could be due to the temporal combination of early melanoma inhibition mediated by T_eff_ plus delayed but durable melanoma inhibition mediated by T_mem_.

### During combinatorial ACT, tumor cell caused by by T_eff_ may be enhanced by IL-2 production from T_mem_

**Fig. 6:**
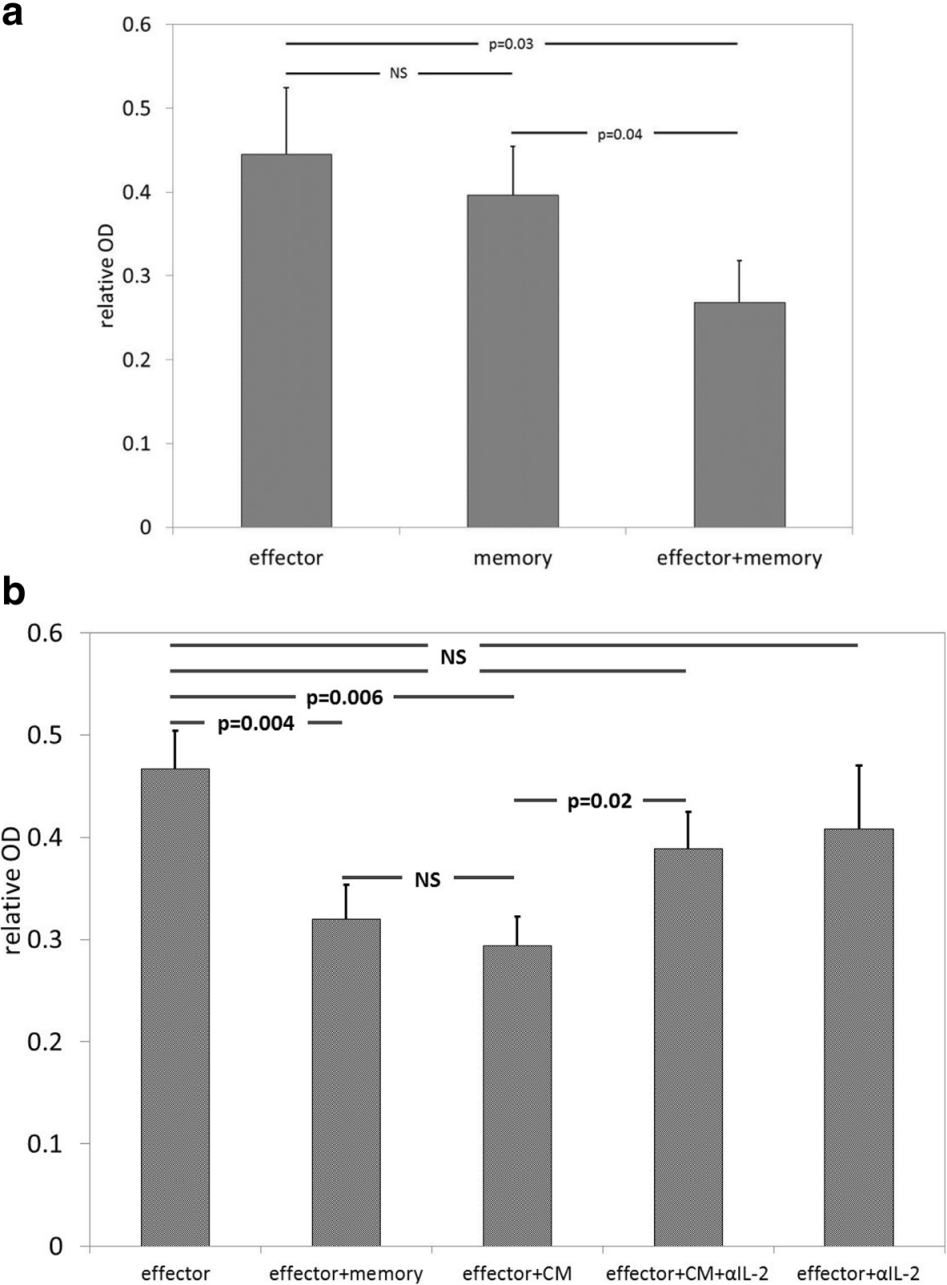
Paracrine release of IL-2 from T_mem_ may potentiate melanoma inhibition by T_eff_. The local interaction of T_eff_ and T_mem_ was examined by performing in vitro melanoma inhibition assays. (**a**) T_eff_ and T_mem_ and T_eff_ + T_mem_ were co-cultured with B16GP33 for 8 h. Although melanoma inhibition was comparable between T_eff_ and T_mem_, significantly stronger inhibition of melanoma was observed with the combination of T_eff_ + T_mem_. (**b**) Conditioned media (CM) was collected from T_mem_ stimulated with GP33 for 5 h. The addition of CM enhanced the efficacy of T_eff_ to a similar degree as the addition of T_mem_. The effect of CM was negated by the addition of anti-IL-2 blocking antibody; the addition of anti-IL-2 blocking antibody alone did not affect the ability of T_eff_ to inhibit melanoma. The addition of anti-IL-2 blocking antibody blunted the ability of T_eff_ + T_mem_ to inhibit melanoma, but it could not be determined if this effect was exerted on T_eff_ or T_mem_ (data not shown). This experiment was repeated twice with similar results, 3 samples per group. (*p*-values as shown on horizontal bars)

To determine if the improved efficacy of combinatorial ACT was attributable to local interactions between cell types, T_eff_, T_mem_, and T_eff_ + T_mem_ were co-cultured with B16GP33 cells in the presence of IL-2 for 8 h. As shown in Fig. 6a, whereas T_eff_ and T_mem_ resulted in comparable levels of melanoma inhibition at this time point, the combination of T_eff_ + T_mem_ resulted in a stronger level of melanoma inhibition. To determine if this enhancement was attributable to paracrine effects, T_eff_ and T_mem_ were separately stimulated with GP33 peptide for 5 h, after which cells were pelleted by centrifugation and conditioned media were collected. As shown in Fig. 6b, the addition of conditioned media from T_mem_ enhanced the ability of T_eff_ to inhibit melanoma to a comparable level seen with T_eff_ + T_mem_. The addition of conditioned media from T_eff_ did not enhance the ability of T_mem_ to inhibit melanoma (data not shown). When anti-IL-2 blocking antibody was added to conditioned media from T_mem_, this effect was lost, suggesting that paracrine release of IL-2 from T_mem_ may potentiate tumor cell death caused by T_eff_. The addition of anti-IL-2 blocking antibody alone did not affect the ability of T_eff_ to inhibit melanoma.

## Discussion

For the first time, we have shown that ACT using a combination of T_eff_ + T_mem_ leads to even more robust control of melanoma growth than ACT using T_eff_ or T_mem_ alone. Our findings suggest that the advantage of combinatorial ACT may result from the induction of stronger infiltration of endogenous CD8+ T cells into tumor, stronger systemic CD8+ T cell responsiveness to tumor antigen, temporal differences in melanoma cell killing, and paracrine IL-2 effects. One recent publication addressed the effects of combining T cell subsets prior to ACT, but this involved co-culturing naïve cells with T_mem_ for 6 days prior to ACT [11]. That approach led to a loss of less-differentiated T cell subsets and impaired in vivo tumor regression [11]. By combining T_eff_ + T_mem_ at the time of adoptive transfer, we were able to retain the innate advantages of each T cell subset.

We observed that combinatorial ACT generated stronger intratumoral CD8+ T cell infiltration than ACT with T_eff_ or T_mem_ alone; this was not due to greater trafficking of adoptively transferred cells into tumor, but a result of more potent induction of endogenous immune responses. This stimulation of ongoing endogenous CD8+ T cell responses may contribute to the heightened efficacy of combinatorial ACT compared to memory ACT. We previously found that combining ACT with checkpoint inhibition led to a similar induction of endogenous TILs that was also associated with greater tumor suppression, stronger systemic T cell responsiveness to tumor antigen, and a more potent long-term anti-tumor immunity [12]. Enhanced melanoma cell death resulting from combinatorial ACT may promote stronger local cytokine/chemokine release, antigen presentation, and priming of endogenous tumor-specific T cells [13], ultimately promoting stronger systemic anti-tumor immunity.

Given the innate predisposition of effector cells to rapidly target antigen, and the ability of memory cells to persist and expand, we hypothesized that the ability of T_eff_ and T_mem_ to engage and inhibit melanoma would exhibit temporal differences. Indeed, in vitro assays demonstrated that T_eff_ cause immediate but transient melanoma inhibition, while T_mem_ cause gradual but persistent inhibition. This pattern is further reflected by the fact that, whereas ACT with T_eff_ resulted in a decline in intratumoral adoptively transferred Ly5.1+/CD8+ T cells at 14 days, ACT with T_mem_ resulted in stable numbers of intratumoral transferred T cells. Thus, the activity of effector and memory cells may be complementary over time, leading to stronger and more prolonged melanoma suppression.

We found that the intrinsic ability of T_eff_ to inhibit melanoma may also be enhanced by local production of IL-2 by T_mem_. Unlike effector T_eff_, T_mem_ are uniquely capable of elaborating IL-2 in response to antigen stimulation [2, 3]. IL-2 supplementation has long been associated with improved outcomes following ACT [14]. Systemic and local delivery of IL-2 in combination with ACT has been shown to increase CD8+ tumor infiltration and suppression of tumor growth [1, 14–17]. With combinatorial ACT, the local, paracrine production of IL-2 by T_mem_ may mitigate the need for exogenous IL-2 administration, decreasing the risk of systemic inflammatory side effects.

By utilizing a melanoma expressing low levels of a viral peptide, we are able to examine the therapeutic effects of bona fide effector and memory differentiation tumor-specific CD8+ T cells in ways not permissible with the use of traditional tumor antigens (e.g., GP100). Although our B16GP33 tumor model does not appear to be more intrinsically immunogenic than parental B16F10 melanoma) [10], further work will be necessary to validate our observations using effector and memory CD8+ T cells directed against naturally occurring melanoma antigens. Our observations suggest that a combinatorial approach to ACT using both T_eff_ and T_mem_ may be a means to optimize the efficacy of adoptive melanoma immunotherapy. While T_eff_ can be readily expanded from TIL cultured in IL-2, it is difficult to prepare large quantities of T_mem_. There is a growing body of literature characterizing the metabolic determinants of effector versus memory CD8+ T cell differentiation, suggesting that it may become feasible to develop this combinatorial approach for clinical evaluation [18–25]. This is an area of ongoing investigation in our laboratory.

## Conclusions

Traditional approaches to adoptive immunotherapy have used effector CD8+ T cells for their rapid proliferative capacity and ability to engage and clear tumor cells. Experimental evidence suggests that memory CD8+ T cells may have physiological advantages over effector CD8+ T cells. Our observations indicate that a combinatorial approach to adoptive immunotherapy using effector and memory CD8+ T cells concurrently results in superior tumor control associated with maximal induction of endogenous T cell responses to tumor. This strategy appears to take advantage of complementary differences in temporal killing of tumor cells by effector and memory CD8+ T cells, as well as local paracrine potentiation of effector CD8+ T cell function by memory T cell-secreted IL-2. These findings suggest that combinatorial approaches to cell-based immunotherapy harnessing diverse states of T cell differentiation may open the door to even greater therapeutic benefit.

## Abbreviations

ACT: Adoptive cell transfer
LCMV: Lymphocytic choriomeningitis virus
T_eff_: Effector CD8+ T cell
TIL: Tumor infiltrating lymphocyte
T_mem_: Memory CD8+ T cell
